# Regions of Chromosome 2A of Bread Wheat (*Triticum aestivum* L.) Associated with Variation in Physiological and Agronomical Traits under Contrasting Water Regimes

**DOI:** 10.3390/plants10051023

**Published:** 2021-05-20

**Authors:** Tatyana A. Pshenichnikova, Svetlana V. Osipova, Olga G. Smirnova, Irina N. Leonova, Marina D. Permyakova, Alexey V. Permyakov, Elena G. Rudikovskaya, Dmitrii K. Konstantinov, Vasiliy V. Verkhoturov, Ulrike Lohwasser, Andreas Börner

**Affiliations:** 1 Institute of Cytology and Genetics SB RAS, 630090 Novosibirsk, Russia; planta@bionet.nsc.ru (O.G.S.); leonova@bionet.nsc.ru (I.N.L.); konstantinov@bionet.nsc.ru (D.K.K.); 2 Siberian Institute of Plant Physiology and Biochemistry SB RAS, 664033 Irkutsk, Russia; svetlanaosipova2@mail.ru (S.V.O.); gluten@sifibr.irk.ru (M.D.P.); Aperm@sifibr.irk.ru (A.V.P.); rudal69@mail.ru (E.G.R.); 3 Faculty of Biology and Soil Science, Irkutsk State University, 664003 Irkutsk, Russia; 4 Institute of Food Engineering and Biotechnology, National Research Irkutsk State Technical University, 664074 Irkutsk, Russia; vvv33@istu.edu; 5 Leibniz Institute of Plant Genetics and Crop Plant Research, 06466 Gatersleben, Germany; lohwasse@ipk-gatersleben.de (U.L.); boerner@ipk-gatersleben.de (A.B.)

**Keywords:** drought tolerance, QTL, GWAS, photosynthesis, antioxidant enzymes, yield components, candidate genes

## Abstract

Understanding the genetic architecture of drought tolerance is of great importance for overcoming the negative impact of drought on wheat yield. Earlier, we discovered the critical role of chromosome 2A for the drought-tolerant status of wheat spring cultivar Saratovskaya 29. A set of 92 single-chromosome recombinant double haploid (SCRDH) lines were obtained in the genetic background of Saratovskaya 29. The lines carry fragments of chromosome 2A from the drought-sensitive cultivar Yanetzkis Probat. The SCRDH lines were used to identify regions on chromosome 2A associated with the manifestation of physiological and agronomical traits under distinct water supply, and to identify candidate genes that may be associated with adaptive gene networks in wheat. Genotyping was done with Illumina Infinium 15k wheat array using 590 SNP markers with 146 markers being polymorphic. In four identified regions of chromosome 2A, 53 out of 58 QTLs associated with physiological and agronomic traits under contrasting water supply were mapped. Thirty-nine candidate genes were identified, of which 18 were transcription factors. The region 73.8–78.1 cM included the largest number of QTLs and candidate genes. The variation in SNPs associated with agronomical and physiological traits revealed among the SCRDH lines may provide useful information for drought related marker-assisted breeding.

## 1. Introduction

Bread wheat (*Triticum aestivum* L.) is one of the most economically and socially important crops in the world. According to FAOSTAT (www.fao.org/faostat/en/#data/QC, accessed on 19 May 2021), approximately 766 million tons of grain were produced on all five continents in 2019. Grain consumers live in 90 countries and account for 40% of the world’s population. Current climate change is contributing to an increase in the frequency and severity of droughts around the world [1,2]. Drought is considered the most challenging stress to global agriculture reducing the annual yield as well as yield stability over the years [3]. A meta-analysis study of 60 publications showed that wheat yields decreased by 27.5% under drought [4]. Wheat cultivation in the dryland was more prone to yield loss than in the non-dryland regions [5].

Understanding the genetic architecture of drought tolerance is of great importance to overcome the negative impact of drought on wheat yield [6]. Many cellular and physiological processes promote drought tolerance [7]. Cell wall re-modelling, the desaturation of membrane lipids, the activation of reactive oxygen species scavengers, the induction of molecular chaperones, and the accumulation of compatible solutes are currently emphasized as the most common and conservative responses of plant cells to drought. These defense mechanisms are combined into complex regulatory networks, including the interaction of various signaling molecules, hormones, and transcription factors [8,9]. The epigenetic mechanisms regulating gene expression also play an important role in plant defense reactions [10,11].

Currently, two methodological approaches are used for identifying QTLs—linkage analysis and associative mapping, which differ in the types of mapping populations and statistical methods. In contrast to linkage analysis, which is mainly used for bi-parental populations, association mapping (AM) is characterized not only by a higher resolution, but also by preliminary information on the candidate genes [12,13]. A combined approach is efficiently used in various plant species to map QTLs that define complex traits [14,15,16].

Both approaches were used for mapping agronomical and physiological traits in the wheat genome under contrasting water supply conditions [17,18,19,20,21]. As a result, dozens of loci associated with variability for physiological parameters, vernalization requirements, duration of phenophases, root traits, and agronomical traits under drought conditions were found in different wheat chromosomes [22]. For some of them, candidate genes were predicted. However, the genetic control of many components of the complex response to drought and the relationship of this response with other physiological reactions of plants are still unknown.

A fully annotated wheat reference genome [23] (https://www.wheatgenome.org/ accessed on 20 April 2021) promotes the use of the QTL approach to study the integral response of wheat to drought by identifying the genes potentially associated with adaptive response. Earlier, using the approach of inter-varietal chromosome substitutions, we discovered a critical role of chromosomes of the second homoeologous group for drought-tolerant status of spring cultivar Saratovskaya 29 (S29) [24]. The most pronounced effect was found in the line with the substitution of chromosome 2A from the variety Yanetzkis Probat. The single chromosome substitution resulted in a dramatic decrease of yield components stability and a decrease in activity of antioxidant enzymes in leaves. The variability for this biochemical trait associated with drought sensitivity was also detected in other substitution lines carrying a single chromosome of the second homoeologous group from different donors [25]. Acuña-Galindo et al. [18] performed a QTL meta-analysis of drought tolerance using 30 QTL studies based on various bi-parental wheat populations and detected 502 loci associated with drought response. The largest number of individual QTLs was located on the chromosomes of homoeologous group 2. A total of 163 QTLs were combined into four clusters included loci associated with different physiological traits, yield and thousand kernel weight. Liu et al. [21] identified five QTL hotspots for yield and related traits under drought and heat stress on chromosome 2A. The researchers identified on chromosome 2A simple sequence repeat markers associated with relative water content [26], chlorophyll fluorescence and osmotic potential [27], thousand grain weight and water-soluble carbohydrates accumulation [28], number of days to heading and maturity [29], and agronomic traits [30,31].

Thus, many studies have shown that chromosome 2A carries a number of QTLs associated with variability for physiological and agronomic traits under drought conditions. These loci were found using genotyped bi-parental mapping populations or association mapping panels of bread wheat. To reveal specific drought-related regions of chromosome 2A we took a different genetic material and used the single chromosome recombinant double haploid lines S29 (YP 2A). The recipient S29 is known as a drought-tolerant Russian cultivar with excellent gluten quality [32]. The high-yielding but drought-sensitive German cultivar Yanetzkis Probat (YP) was the donor of chromosome 2A. Only recombinations for chromosome 2A determine the diversity of drought tolerance in this hybrid population. The genotyped recombinant lines were phenotyped according to a complex of physiological and biochemical characteristics and agronomic traits. The physiological and biochemical characteristics included photosynthetic gas exchange and chlorophyll fluorescence parameters, determining the status of the photosynthetic apparatus [33], lipoxygenase activity, a key enzyme in the synthesis of biologically active oxylipins including phytohormone jasmonic acid [34], catalase activity, which controls the safe H_2_O_2_ level in cells [35], and the activity of four ascorbate-glutathione cycle enzymes. The latter provide a powerful mechanism that protects the photosynthetic apparatus from oxidative damage [36]. The studied agronomical traits included yield and developmental parameters.

The purpose of this study was to identify the regions on chromosome 2A of bread wheat associated with the manifestation of physiological and agronomical traits under contrasting water supply conditions, and to identify candidate genes in these regions that may be associated with adaptive gene networks in wheat. The localization of QTLs was carried out using combined linkage analysis and association mapping.

## 2. Results

### 2.1. Trait Variation between S29 and YP and among the SCRDH Lines

The trait values of the parental and SCRDH lines that were measured in climatic chamber, greenhouse, and field experiments are presented in Table S1. The genotypes were characterized by mean values, standard deviations, limits of trait variations, ratios of maximum values to minimum values and H^2^. Out of 38 characters studied, 17 were related to agronomical traits, and 21 to physiological and biochemical traits, respectively. Under both irrigation conditions, the donor YP showed earlier tillering but later ripening, shorter stem and peduncle length, longer main spikes, greater number of spikelets, smaller grains, and a lower TGW compared to the recipient S29. Under normal watering, YP was more productive than S29. Under drought, YP formed more grains from the plant than S29 but their weight was comparable to that of the recipient. The two cultivars did not differ in photosynthetic parameters. The donor of chromosome 2A showed a higher WUE under watering and lower under drought compared to the recipient. F_0_ was approximately the same in YP under both irrigation conditions and significantly increased in S29 under drought. The recipient showed higher ChlA and Car content, and LOX activity under both irrigation conditions.

A high heritability was demonstrated by photosynthetic and chlorophyll fluorescence traits while for grain number and weight it was the lowest (Table S1). In most cases, the traits showed transgressive variation indicating their polygenic control. Photosynthetic traits showed the largest variability among the lines under both watering conditions. Thus, transpiration rate and stomatal conductance differed 10 times between minimal and maximal values among the lines. The activity of antioxidant enzymes (DGAR, GR, CAT) and LOX activity varied by 5–9 times. Under watering, two-fold differences were found between the maximum and minimum values of the productivity in secondary tillers and of the whole plant. These differences increased under drought and became three-fold (Table S1).

The variability of the tolerance indexes is similar to the variability of the traits under drought conditions (Table S2). The largest range and max/min ratio demonstrated the tolerance indexes for gas exchange parameters (Gs, E, A), GR, and LOX with yield components and pigment content being the next. An analysis of variance showed that genotypic differences for every studied trait among 92 lines were statistically significant (data not shown).

Three principal components (PCs) described the variability in SCRDH population in each of the two irrigation conditions (Table 1, Figure S1). In total, PCs explained 74.4% and 80.4% of the variability under normal watering and drought conditions, respectively. More than 40% of the total variability under normal watering was determined by PC1, and G_S_ was the main contributor. LOX, DT, and WUE were next. Under drought, about 38% of variation was determined by PC1 with LOX as the main source of variability followed by DT, TGW, G_S_, and SpkN. PC2 contributed about 17% to the total variation under normal watering. LOX, ETR and F_0_ were the main source of variability. Under water deficit, 27% of variability was determined by PC2 with DT and Gs as the main contributors of variation. About 15% of variation was determined by PC3 under both irrigation conditions. DT, GNsecond, GNtotal, and GC were the main contributors under normal watering. Under drought, TGW, Gs, GNsecond, GNtotal, and LOX gave the largest input in PC3. Thus, Gs, LOX, DT, and TGW were the main factors determining the variability of physiological and agronomical traits in the population of SCRDH S29 (YP 2A).

For the tolerance index, the total variance explained by the three main components did not exceed 60% (Table 1). PC1, with the largest contribution (~40%), was associated mostly with tolerance indices for agronomical traits and gas exchange parameters. PC2 and PC3 were determined mainly by the traits characterizing photosystem II and pigment content in the leaves.

### 2.2. Clustering of SCRDH Lines

Cluster analysis was used for grouping the lines according to their general phenotypic variability. The lines formed four main clusters under both irrigation conditions (Figure S2). The average trait values of the entire population of SCRDH lines (Table S1) were compared with the average values in the individual clusters. The results are presented in Table 2. Under normal conditions, the grouping of the lines was largely associated with gas exchange parameters. In the first and forth clusters, the lines showed a contrasting gas exchange parameter without impact on yield components. The lines from the second cluster were characterized by low gas exchange parameters resulted in lowered yield components. Additionally, they had a lowered LOX activity. The lines of the third cluster showed an increase in chlorophyll content and a doubled LOX activity. Under water deficit gas exchange parameters varied in three of four clusters. LOX was the next important trait, which participated in grouping of the lines in all clusters, and its activity both increased and decreased. Total plant productivity variated mainly due to the grain number and weight of the secondary tillers under both watering conditions.

Physiological and biochemical traits: A: photosynthetic rate (μmol m^−2^ s^−1^); Car: carotenoids; ChlA,B: chlorophyll A,B (mg/g of dry leaf weight); E: transpiration rate (mmol m^−2^ s^−1^); WUE: water use efficiency as net photosynthesis/transpiration; ETR: maximum electron transport rate (photon m^−2^ s^−1^); F_0_: basic chlorophyll fluorescence yield; Fv/Fm: maximum quantum yield of PSII photochemistry; NPQ: non-photochemical quenching; Gs: stomatal conductance (mol m^−2^ s^−1^); SW: fresh weight of the main shoot (g); Y(II): effective photochemical quantum yield of photosystem II; CAT, DHAR, GR, SOD, LOX: activity of catalase, dehydroascorbate reductase, glutathione reductase, superoxide dismutase and lipoxygenase (U/mg protein).

### 2.3. Trait Correlations

**Figure 1:**
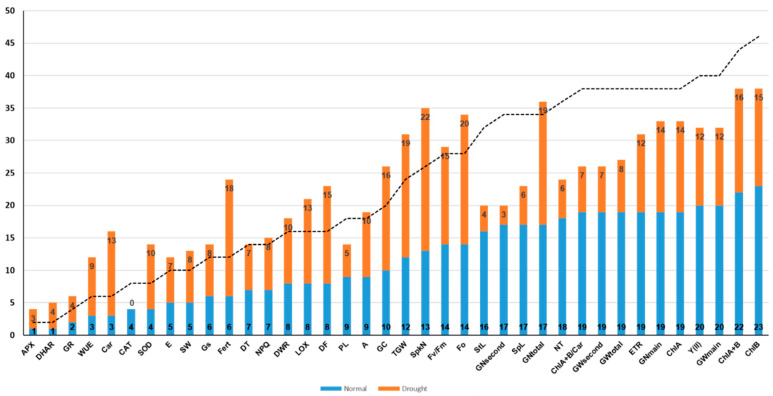
The number of correlations of each trait on irrigation (blue) and drought (orange). Dotted line is a formal line showing the number of correlations on drought equal to their number on irrigation. The number of correlations for the trait on drought increases if the bar is located above the dotted line. The number of correlations for the trait on drought decreases if the bar of the diagram is located below the dotted line.

Correlation analysis (Table S3) showed that an almost equal number of reliable correlations between the traits were formed during normal irrigation and during drought, 210 and 209, respectively. At the same time, during drought, the number of positive relationships decreased from 167 to 117, and the number of negative ones increased from 53 to 92. The traits were arranged in ascending order of the number of correlations during normal irrigation (Figure 1). All the traits can be divided into three groups according to the number of correlations found. APX, DHAR, GR, Car, and SOD had the smallest number of correlations under irrigation, from 1 to 4, but the number of correlations for these traits increased 2–4 times under drought. CAT was the only exception in this group for which no correlations were found under drought. The largest number of correlations under normal irrigation, from 16 to 23, had agronomical traits StL and NT, yield components GNsecond, GNtotal, GWsecond, GWtotal, GNmain, as well as ChlA, ChlB, ChlA+B, ChlA+B/Car, ETR, and Y(II). For all these traits, with the exception of GNtotal, the number of correlations decreased under drought. Other traits (with the exception of PL) had an average number of correlations from 5 to 14 in both conditions. Among physiological features, a three-fold increase was observed for WUE and Fert, but among leaf pigments, a four-fold increase was observed only for Car. Among the developmental traits, the correlations for DF almost doubled that under drought. This increase was due to the emergence of novel correlations between DF and both content of leaf pigments and parameters of photosynthesis. Most of the productivity components reduced the number of correlations on drought. The increase was noted only for TGW, Fert and SpkN. GC was characterized by a similar variability. Among the biochemical traits, the antioxidant defense enzyme SOD showed the greatest correlation dynamics. It increased the number of significant correlations under drought conditions by 2.5 times. A large number of correlations were revealed for LOX both during irrigation and during drought. In the latter case, their number well increased (Figure 1). In general, the yield components correlated largely with the pigment content and chlorophyll fluorescence parameters than with the gas exchange parameters. GC in grain negatively correlated with all yield components in normal watering. However, under drought, this trait showed a positive relationship with the number of grains of secondary tillers and the total number of grains per plant, as well as with all developmental traits (Table S3).

### 2.4. QTL Analyses

**Figure 2:**
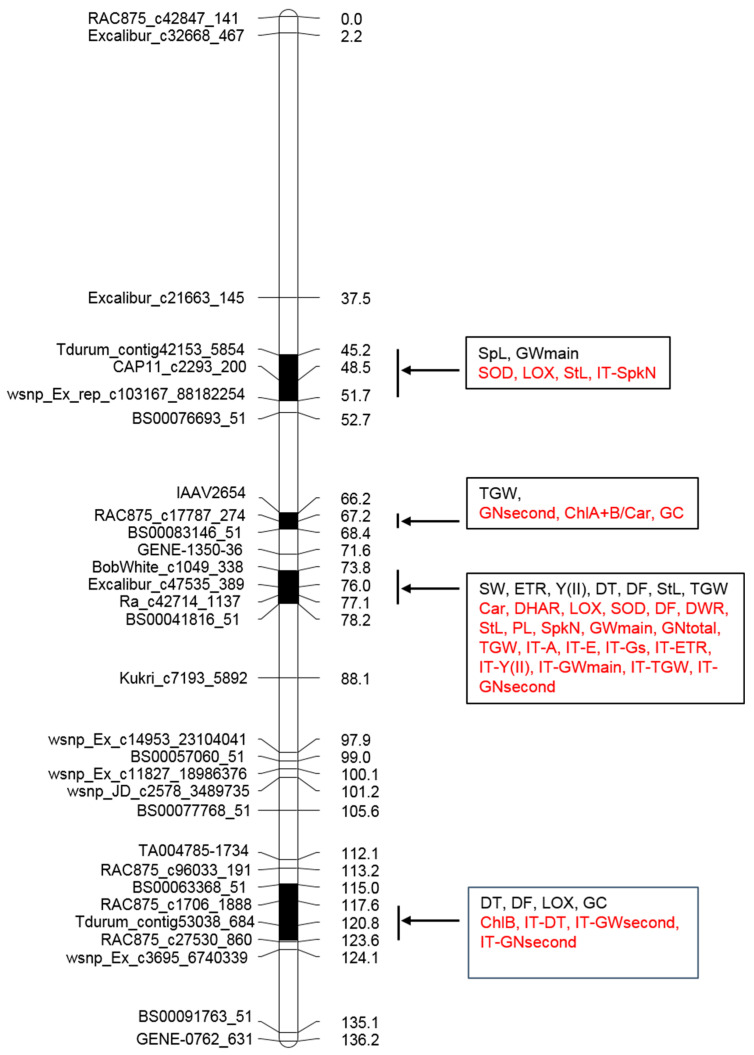
Genetic map of chromosome 2A showing QTL clusters for physiological and agronomical traits in SCRDH population S29 (YP 2A). Skeleton SNP markers are shown on the left, Kosambi distances (cM) are on the right side of the chromosome. The loci mapped under drought conditions are shown in red, under watering—in black.

Two experimental models (linkage analysis and association mapping) were used to search for the QTLs. For linkage analysis, 146 polymorphic SNP markers were used, mapping to chromosome 2A of wheat (https://wheat.triticeaetoolbox.org/ accessed on 1 June 2020). To construct a genetic map, a threshold (LOD) > 2.0 was applied in MultiPoint programs. SNP markers were clustered into two linkage groups consisting of 143 and 3 markers, respectively. Thirty SNP markers out of 143 were used as skeleton markers; the length of the linkage group was 136.2 cM (Figure 2). It was noted that the order of the markers did not differ significantly from the consensus map of wheat chromosome 2A [37].

Using the linkage model, 19 QTLs were found on chromosome 2A with LOD >2–3. Seven of them were detected in the region of 123–124 cM and grouped around the marker RAC875_c27530_860. They were associated with SpkN, ChlB and ChA+B under drought; GNmain under watering and tolerance indexes IT-GWtotal, IT-GNtotal, and IT-LOX. Another cluster was found in the region 73–78 cM of the genetic map. It included QTLs for StL, TGW, Fert, GNmain, GWmain, and SpL under normal watering. Two-QTL clusters were detected at the position 134–135 cM (for DF under drought and GR under watering) and at the position 66 cM (for NT under drought and IT-NT). A single QTL for IT-NT was found at position 48.5 cM.

The number of QTLs detected by the linkage analysis was a relatively low for the 38 traits showing a wide range of phenotypic variation and multiple interdependencies. For this reason, the association mapping approach was applied to find as many associations as possible between SNP allelism and trait variability. This approach is known to be more precise and may be applied to any population or collection of lines [38].

To analyze the population structure (Q-matrix), 146 polymorphic SNPs located on the chromosome 2A were used. The number of putative genetic clusters according to the STRUCTURE program was five, while the sub-clusters significantly differed in the number of included SCRDH lines (Figure S3). The highest expected heterozygosity was observed in clusters 2 (0.0604) and 3 (0.1053) relative to clusters 1, 4 and 5 (0.0307, 0.0283 and 0.0025, respectively).

Using association mapping approach, 58 QTLs were identified on chromosome 2A. Sixteen of them were associated with physiological and biochemical traits, 28 with agronomical, and 14 with ITs. Five marker-trait associations (MTAs) demonstrated the significance at *p* < 0.001, more than 40 associations—at *p* < 0.01, and more than 30 at *p* < 0.05. The MTAs with the significance *p* < 0.01 or higher are presented in Table 3.

QTLs for physiological and biochemical traits showed a phenotypic variation range of 4–10%. Eight of them manifested at normal watering and eight manifested under drought. Eight loci were detected for antioxidant enzymes and five for pigment content. For chlorophyll fluorescence parameters ETR, Y(II) and their ITs, 4 QTLs were found. No associations with a confidence level *p* < 0.001were found for the gas exchange parameters A, E, and G_S_, while the loci for their ITs were mapped. For agronomical traits, 13 QTLs were found under normal watering, 12 under drought, and 3—under field conditions (Table 3). Their PVEs ranged from 4 to 15%. QTLs were found for all developmental traits. QTLs for DF were mapped under all irrigation conditions. MTAs for DF under field conditions and for DWR under drought achieved the highest significance level (*p* < 0.001). The most QTLs (7) were found for StL under both irrigation conditions, with the highest significance detected for MTA in the field condition. The next for the number of QTLs found was TGW, for which 5 trait QTLs were found under both irrigation conditions and 1 IT QTL. One of the MTAs for TGW achieved *p*-value < 0.001. Two QTLs were found for SpL, and for PL. Single QTLs were found for such component of plant productivity as GNmain, GWmain, GNsecond, and GNtotal. QTLs for IT-GWmain, IT-GNsecond, IT-GWsecond, and IT-GNtotal were also identified. For GC two QTLs were found, one for each watering condition. The calculation of the additive effects of SNP alleles was carried out with respect to the allele of the recipient cultivar S29. Both positive and negative additive QTL effects were detected. For certain trait, these effects could be oppositely directed. For example, three QTLs were identified for TGW under irrigation. For two of them, a positive additive effect was observed, and for one, a negative one. For yield components, only negative additive effects were observed.

### 2.5. QTL Clustering

Only 5 out of 58 QTLs with a declared significance level were single (Table 3). Others were grouped together in five regions along chromosome 2A. They included different number of QTLs within the regions of different length. The distribution of the four clusters along the genetic map of chromosome 2A is presented in Figure 2. The proximal cluster, associated with SNP marker BobWhite_c13373_250, could not be located on the genetic map. On the consensus map, this marker was extremely positioned on the short arm of chromosome 2A. This cluster included QTLs for ChlA content, ChlA+B content and SpL under normal watering, and for IT-SpL under drought. Since this cluster could not be mapped, it was not included in the further study. The next cluster was located between SNP markers Tdurum_contig42153_5854 and wsnp_Ex_rep_c103167_88182254 on the position 45.2–51.7 cM. It included QTLs for SpL and GWmain under normal watering, and QTLs for SOD, LOX, StL and IT-SpkN under drought. The next cluster within the region of 66.2–68.3 cM, located between SNP markers IAAV2654 and BS00083146_51, combined QTL for TGW under normal irrigation and QTLs for GC, GNsecond and ChlA+B/Car under drought. The next cluster in the region of 73.8–78.1 cM was positioned between SNP markers BobWhite_c1049_338 and BS00041816_51. It was the most numerous. It was characterized by a sharp increase in the number of QTLs under drought in addition to those, which were detected under normal watering. In this region, three QTLs for physiological traits SW, ETR, Y(II), and four QTLs for agronomical traits DT, DF, StL, and TGW were detected under watering. Under drought, the number of QTLs increased to 20. QTLs for DHAR, LOX, SOD activity and carotenoid content were found in the cluster. This group again included developmental and agronomical traits as well as ITs for all photosynthetic and for several agronomical traits (Figure 2). The most distal cluster was detected in the region of 117.6–123.0 cM between SNP markers BS00063368_51and RAC875_c27530_860. Under normal irrigation, it included QTLs for DF, LOX, and GC. Under drought, it included QTLs for ChlB content, IT-DT, IT-GNsecond and IT-GWsecond.

### 2.6. Candidate Gene Selection

The search for the candidate genes associated with responses to drought was carried out in the regions, which were marked by SNPs that were reliably associated with the phenotypic manifestation of the studied traits in SCRDH lines. The following regions of chromosome 2A were analyzed: 45.2–52.8 cM (region 1), 66.2–68.3 cM (region 2), 73.8–78.1 cM (region 3), and 117.6–123.0 cM (region 4) (Table 4). A total of 1,094 genes were found in these regions. According to the constructed genetic map, the length of the target regions ranged from 2.1 to 6.5 cM (Table 4). The regions differed significantly both in the total number of the found genes and in the number of selected candidate genes. Region 2 was three times smaller than region 1, but it contained a larger number of genes, and the calculated gene density by 1 cM in this was several times higher. Region 3 was comparable in size to region 1, but contained almost 4 times more genes, and the estimated gene density was the highest here. Among all the genes found in the regions, those genes were selected that corresponded to the GO terms associated with the photosynthetic apparatus, response to oxidative stress, and lipid metabolism during drought. Thirty-nine candidate genes were selected: 4, 9, 17, and 9 genes in each of the four above-mentioned regions, respectively (Table 4). The largest number of selected genes were associated with the general response to drought (10 genes) and the activity of the stomatal apparatus (13 genes). Of the 39 genes, 17 were identified as transcription factors (TFs). Regarding the function of the identified candidate genes, the first region contained 3 structural genes associated with various metabolic processes and 1 TF (Table S4). In the second region, 7 out of 9 genes were the genes for TFs. Some of TFs are related to processes of development and adaptation to stress, for example, BZIP TF (homologue of *AT2G41070*) and WRKY TFs (homologues of *AT3G56400* and *AT2G46400*), which are involved in plant protection from various stresses by regulating jasmonate and salicylate metabolic pathways. The candidate genes found in the region 3, where they are most abundant, showed the greatest functional diversity. Only 5 out of 17 genes were TFs; some of them were drought induced. Most of the remaining genes were associated with the formation and functioning of stomata and the processes of photosynthesis. Two of them belonged to the lipase/lipoxygenase family, activated by stresses, in particular, by heat. In the distal region 4 of chromosome 2A, four out of 9 genes were transcriptional factors involved in abscisic acid and auxin signaling. Remaining genes were associated with response to various types of stress, including oxidative stress, as well as with biosynthesis of flavonoids and development of flower meristems (Table S4).

## 3. Discussion

Plants implement various mechanisms of drought tolerance. These mechanisms can be specific to species adapted to different geographic and ecological conditions. Winter and spring wheat cultivars from various regions of origin and cultivation differ significantly in the level of drought tolerance and types of responses to water deficit. The spring cultivar S29 obtained in the arid steppe climate of the Volga region is distinguished by a high drought tolerance [32]. The high drought tolerance of S29 is due to the morphological adaptations, such as narrow leaves with dense pubescence and due to a high viability of the photosynthetic apparatus at the end of the growing season [32]. The latter may be related with the “stay-green” effect [25]. The latest data showed that S29 has a constitutive tolerance to water deficiency at the cellular level. For S29, the main criteria of a stress state, such as antioxidant mechanisms activation as well as of an increase in the proline or soluble sugars content under water scarcity are not characteristic [25]. Our earlier evidence showing the importance of chromosome 2A for drought tolerance [24] motivated the present study. In this work, we showed that chromosome 2A substitutions was associated with a decrease in antioxidant potential. For the first time, we used SCRDH lines S29 (YP 2A) in order to identify the chromosomal regions and the possible genetic factors responsible for the adaptive mechanisms. The phenotypic diversity in this population is determined only by the recombination between chromosomes 2A of drought tolerant and drought sensitive cultivars S29 and YP, respectively.

The largest intra-population differences (max/min ratio) were observed for gas exchange parameters, both under normal watering and drought conditions (Table S1). Gs was a classifier in all components of the PC analysis (except PC3 under normal watering) (Table 1). In cluster analysis (Table 2), the lines segregated into groups mainly according to gas exchange parameters. This may indicate the genetic conditionality of these differences between the lines. Under irrigation, the group of gas exchange traits Gs, E, and A did not reveal close and systematic correlations with other studied traits. However, under drought, all three parameters correlated with the efficiency of electron transport in photosystem II, GR, and TGW. This may indicate a coordinated regulation of the manifestation of these traits during drought.

LOX was the second important classifier in phenotypic analysis. The recipient and donor cultivars differed significantly for this trait under both irrigation conditions (Table S1). This trait made a significant contribution to the same principal components as photosynthetic parameters. In cluster analysis, two clusters adversely differed in LOX activity under normal watering; under drought, this trait varied noticeably in all four clusters (Table 2). This trait showed the largest number of correlations with the studied traits compared with other enzymes under both water regimes. Under irrigation, LOX mainly correlated with the parameters of chlorophyll fluorescence. Under drought, the value of correlations increased, and correlations with developmental traits DF and DWR, fertility, chlorophyll A and B content, and gluten content in grain were added to them. The donor cultivar YP exceeded S29 for yield under irrigation and drought (Table S1). The differences in the final productivity of the SCRDH lines were due to the variability of the yield components of secondary tillers, which formed the grain after the onset of water deficit. Under drought, the range of variation between maximal and minimal values for grain number and weight was 3–4 times. The same traits showed a large variability for their tolerance indexes (Table S2). The weight and number of grains of secondary tillers and the whole plant contributed to PC3 under both irrigation conditions. When clustering the lines on irrigation, a decrease in these traits was associated with a decrease in gas exchange parameters in one of the clusters. This was not observed under drought. On irrigation, the yield components were positively correlated with chlorophyll content, however, on drought, the correlations were negative. In addition, these traits changed their dependence from the activity of antioxidant enzymes. Under irrigation, the correlations were positive with CAT, GR, and LOX whereas under drought new negative correlations with SOD, DHAR, and LOX appeared. The recipient cultivar maintained a high TGW under both watering conditions. The latter trait made a large contribution to the diversity of PC1 and PC3 in drought. During drought, the number of correlations for this trait increased significantly and their composition changed. Under drought conditions, TGW correlated not with yield components, but with the parameters of photosynthesis and chlorophyll fluorescence. Thus, on the genetic background of S29 the recombination between the two homologous chromosomes 2A resulted in phenotypic variability for gas exchange, LOX activity, secondary tillers yield, and total yield, as well as TGW. Variability was also found for the content of leaf pigments and for the parameters of chlorophyll fluorescence.

The experimental QTL search model based on the constructed genetic map of the population revealed two multi-locus regions of chromosome 2A. They grouped six or seven QTLs, mainly related to yield components. The rest of the positions contained 1–2 loci. This model was not satisfactory enough to explain the phenotypic diversity and correlations between 38 studied traits and their ITs. In addition, this model did not allow the identification of loci associated with physiological and biochemical characteristics. Using the association mapping approach, which is usually applied to association panels of wheat genotypes, turned out to be more productive. This approach made it possible to identify 58 QTLs, the vast majority of which were grouped into 4 clusters with the number of loci from 4 to 17. The size of the regions of chromosomes 2A, in which the clusters were located, ranged from 2.1 to 6.5 cM according to the genetic map. Previously, four meta-QTL clusters on the short and long arms of this chromosome were identified [18]. Clusters saturated with QTLs were identified in a bi-parental bread wheat mapping population [21] and in association mapping panels of bread and durum wheat [30,39].

The largest number of QTLs, six, was found for StL, of which 4 were found in the region with a size of 1.1 cM (Table 3). Earlier, a similar trait—plant height—was mapped on the short and long arms of chromosome 2A using microsatellite and SNP markers [21,26,29]. The next most common QTL was for TGW. Multiple QTLs for this trait on chromosome 2A were previously identified (21,25,30]. The agronomic significance of this trait is even higher than for the number of grains, since maintaining a large grain size during drought makes it possible to form marketable grain. Of the six identified QTLs (2—on irrigation, 3—on drought, and IT-TGW), 4 were found in the same cluster and were associated with the same closely linked SNP markers Ra_c42714_1137 and Ku_c5710_312 (Table 3). It was with these two markers that 18 associations were identified with both agronomic (StL, TGW, developmental traits, grain number, and their ITs) and physiological and biochemical traits and their ITs (SW, DHAR, IT-A, IT-E, IT-Gs). The linked SNP markers BS00041816_51 and Ra_c34214_1320 associated with ETR, Y(II) and their ITs are located only 1cM away from Ra_c42714_1137 and Ku_c5710_312 markers.

The genotype profiling of SCRDH lines was done with a 15k wheat array obtained based on 90k chipset [37]. The latter was previously used for MTAs search in the North American Spring Wheat (SW-AM) and Hard Winter Wheat (HWW) association mapping panels. We compared the associations of the four SNP markers mentioned above with MTAs found in these panels using T3/Wheat database (https://triticeaetoolbox.org/wheat/view.php?table=markers&uid=100822 accessed on 17 March 2020). The SNP marker Ra_c34214_1320 was associated with grain yield and canopy temperature in SW-AM panel and with ETR, Y(II) and their ITs in our set of lines. The marker Ra_c42714_1137 in both panels was associated with flowering date and chlorophyll content in leaves. The adjacent marker Excalibur_c28017_641, situated in the same cluster, in the HWW panel was associated with the mentioned physiological traits and with many yield components in the two sets of genotypes. In our experiment, this marker was associated with CAR, DT and GWmain. The fourth cluster on the SCRDH genetic map included SNP marker BS00065245_51 that in SW-AM panel was associated with chlorophyll content, several yield components and protein content in grain. In our set of the lines, the marker was associated with IT-GWsecond, GC and LOX. GC is known to be the main component of grain protein. Earlier, Permyakova et al. [40] showed a strong positive correlation between LOX and gluten content in grain. At the same cluster, QTLs for ChlB, DF, IT-DT, and IT-GNtotal were found in our experiment.

In our study, we mapped QTLs associated with the activity of antioxidant enzymes and lipoxygenase, which are important components of the protective response. For CAT, GR, and DHAR, these were single loci. For SOD and LOX, two and three loci were identified, respectively (Table 3). DHAR, SOD, and LOX were co-localized in the certain clusters (Figure 2). It should be noted that these three enzymes showed an increase in correlations with other phenotypic traits under drought (Figure 2). Most of QTLs for their activity were revealed under water stress conditions. This may indicate the activation of antioxidant defense pathways during drought. In general, the detection of a larger number of QTLs during drought than during irrigation may indicate the existence of regulatory genes for a general response to drought on chromosome 2A, which associate the process of photosynthesis with the accumulation of biomass, productivity, and plant development both under irrigation and water stress.

Bioinformatic analysis of the four target regions 1–4 (Table 4) including QTL clusters differ both in the total number of genes and in the number of selected candidate genes (Table 4). Complete information about identified genes is presented in Table S4. Almost half of them were transcriptional factors (TF), and they were discovered in all the four regions of chromosome 2A. Considering that the number of identified QTLs in each region increased under drought, this indicates their important role in adaptation to drought. Eleven of these TFs belonged to the five multigene families of transcription factors AP2/EREBR, bZIP, MYB, NAC, and WRKY. Their role in abscisic acid (ABA) dependent and independent pathways of response to drought has been well studied in plants [41], including wheat [42].

In the region 1, 45.2–52.8 cM, the gene *TraesCS2A01G066700* was found to be a homolog of Arabidopsis gene belonging to the Ribulose bisphosphate carboxylase small chain gene (RBCS) family. Rubisco plays a major role in the assimilation of CO_2_ during photosynthesis and involves inorganic carbon in the biological cycle of the synthesis of organic matter synthesis and plant organ building. Thus, the gene *TraesCS2A01G066700* can reasonably be associated with the identification of QTLs for individual components of plant productivity in this region. Recently, Qin et al. [43] through genomic studies have shown that the *RBCS* gene family in wheat is located on the short arms of the chromosomes of the second homoeologous group. Expression of *TaRBCSs* markedly decreased under the influence of drought. In most of the *TaRBCSs* promoters, MYB binding sites were found [43], which indicates the regulation of the expression of this gene by transcription factors of the MYB family. Qin et al. [43] noted the need to study the molecular mechanism of regulation of *TaRBCS* expression under drought conditions, since the accumulation and catalytic efficiency of Rubisco could be controlled by regulation of *TaRBCS* expression. The gene *TraesCS2A01G069300*, a homologue of *MYB36* of Arabidopsis, presumably corresponds to *TaMYB25* detected by the genome-wide analysis of *MYBs* in bread wheat [44]. The expression of this factor in leaves and interactions between Myb36 and proteins involved in responses to drought (peroxidases, dehydrins, a protein necessary for the regulation of stomatal opening by auxin, ABA, and external Ca2^+^) were shown.

In the region 2, 66.2–68.3 cM, the gene *TraesCS2A01G099900* is a homologue of the TF genes of the EIN3/EIL family, which plays a key role in the ethylene signaling pathway, and the GRAS family of the DELLA subfamily, which is involved in hormonal signaling pathways mediated by gibberellic, jasmonic, abscisic, salicylic acids and ethylene. Genome-wide analyzes of these families in *T. aestivum* showed that chromosome 2A carries two genes of EIN3/EIL family [45] and 10 genes of the GRAS family [46]. The gene *TraesCS2A01G099400* can be considered as a candidate controlling the trait TGW. *TraesCS2A01G099400* encodes a bZIP homologue of TF ABI5 in Arabidopsis, which is a global regulator of seed maturation responsible for the accumulation of storage substances [47]. In wheat, it may be responsible for the grain size, which determines TGW. In the same cluster, homologous genes coding TF ORE1 (*TraesCS2A01G101400*) and two functionally similar ones, TFs WRKY46 and WRKY7070 (*TraesCS2A01G104900* and *TraesCS2A01G104800*) were found. ORE1 plays a central role in the positive control of leaf senescence in Arabidopsis [48]. Two other TFs are involved in the brassinosteroid pathway of plant growth regulation and drought tolerance [49]. It is possible that their interaction under drought conditions leads to an increased accumulation of gluten in grain, which made it possible to map QTL for GC under these conditions in the region 2.

The region 3, 73.8–78.1 cM, flanked by SNP markers wsnp_Ex_c2337_4379619 and BS00041816_51, where the largest number of MTAs was detected, turned out to be the most saturated with candidate genes. The MTAs can be divided into several groups. One of these included QTLs associated with developmental traits. They can be associated with the two candidate genes, *TraesCS2A01G347000* and *TraesCS2A01G337900*. The first of them is a homologue of the Arabidopsis gene of the *FLOWERING LOCUS T* (*PEBP*) family, which is expressed in leaves and promotes the transition to the flowering phase [50]. Its numerous functions also include regulation of stomatal opening [51]. As gas exchange is dependent from stomatal movement, QTL localization for IT-A and IT-E may be associated with PEBP. The second candidate gene is a homologue of the MADS-box TF AGAMOUS-like 16, which negatively regulates the flowering time transition through FLOWERING LOCUS T [52]. It also participates in the formation of stomatal complexes [53].

Another group of candidate genes in region 3 is associated with various aspects of photosynthetic processes. The gene *TraesCS2A01G342600* is a homologue of gene *Lhcb6*, which encodes a protein that is part of the light harvesting complex. It captures photons and transfers energy to the reaction center of the chlorophyll molecule [54]. At the same time, it has a protective function against reactive oxygen species. The gene *TraesCS2A01G344400* turned out to be a homologue of the *PsbQ-like1* gene, which synthesizes a protein included in the chloroplast NAD(P)H dehydrogenase complex under stress and supports the transfer of electrons to photosystem II [55]. These genes can be associated with the variability in the parameters of chlorophyll fluorescence ETR and Y(II) and their ITs. Several candidate genes were also found that are related to the formation of the stomatal apparatus and indirectly affect the processes of gas exchange. These are the genes *TraesCS2A01G343000*, a homologue of the epidermal patterning factor gene involved in the formation of stomata and their frequency [56], as well as the *TraesCS2A01G357100.1* gene, a homologue of the protein gene from the Leucine-rich repeat (LRR) family, which determines the symmetric pattern of stomata [57]. The gene *TraesCS2A01G340000* was found to be homologous to the *Cryptochrome 1* gene, which regulates stomatal conductance under high irradiation and is controlled by ABA levels [58]. These genes can be associated with the variability for the chlorophyll fluorescence parameters ETR and (YII) and their ITs. Several candidate genes were also found that are related to the formation of the stomatal apparatus and indirectly affect the processes of gas exchange. The gene *TraesCS2A01G350000* being a homolog of Arabidopsis gene for Nitrate transporter 1.2 may also be involved into photosynthetic processes since this gene is a mediator and transporter of ABA for the regulation of stomatal aperture in inflorescence stems [59]. In the same region, the *TraesCS2A01G350000* gene was identified, which is a homologue of the mitochondrial pyruvate carrier gene, being a negative regulator of ABA signaling in stomatal guard cells in response to drought. It can be assumed that all the four candidate genes are involved in the regulation of photosynthesis under conditions of water stress and can be associated with QTLs for IT-A and IT-E. It should be noted that suggestive QTLs for these traits and were mapped in the region 77–78 cM (data not shown). The variability in the expression of candidate genes associated with photosynthetic parameters can be relevant to different rates of metabolic processes and accumulation of organic substances. This ultimately leads to differences in the formation of productivity components among SCRDH lines and clustering of the corresponding QTLs in the same region with photosynthetic QTLs. In the region 3, the gene *TraesCS2A01G364200* was found, a homologue of one of the six general TF, which, along with RNAP II and MEDIATOR, are the main elements required for initiation and maintenance of gene transcription [60].

We found many phenotypic correlations between LOX and agronomic and physiological-biochemical traits (Table 1 and Table S3). Only in the region of 73.8–78.1 cM were the candidate genes associated with LOX activity found. These were the genes *TraesCS2A01G342100*, which is a homologue of the Lipoxygenase domain-containing 1 (PLAT-domain) gene family, the *TraesCS2A01G364500* gene encoding lipase, and the *TraesCS2A01G331300* gene belonging to the FATTY ACID EXPORT 1 (FAX1) lipid metabolism gene family. *PLAT1* represents a downstream target of ABA signaling pathway functioning as a positive regulator of abiotic stress tolerance. It also is involved in regulating plant growth, and in general could be promising targets for improving abiotic stress tolerance without yield penalty [61]. Expression of the second gene is heat-induced [62]. The *FAX1* gene is involved in lipid and fatty acid homeostasis, and its function is crucial for biomass production [63]. The gene *TraesCS2A01G367700*, a homologue of the Arabidopsis genes from the caleosins family is also associated with LOX activity. Caleosins are involved in metabolism of oxylipins, and their expression is induced by abiotic stresses, including drought [64]. By participating in the regulation of stomatal movements, caleosins modulate drought tolerance.

In the position 77.08 cM of the region 3, in link with SNP markers Ra_c42714_1137 and Ku_c5710_312, QTLs for TGW were mapped under irrigation and drought. A candidate gene for this trait may be *TraesCS2A01G354300*, which is a homologue of the NAC domain containing protein 3 TF [65]. It controls the process of leaf senescence and is a paralog of the transcription factor ORE1, a homologue of which was found in the region of 66.2–68.3 cM. The trait TGW forms in the final stages of plant development. Therefore, it depends on the intensity of metabolic flows from leaves to storage organs, which, in turn, could be determined by the rate of the senescence process.

In the region 4, 117.6–123.0 cM (Table 4), the gene *TraesCS2A01G536600* was identified, which also turned out to be a homologue of FLOWERING LOCUS T [52]. It may be associated with QTLs for developmental traits and variability in productivity parameters under the influence of drought. Homologous genes of heme oxygenase (*TraesCS2A01G533900*) and calreticulin (*TraesCS2A01G545600*) were also found in this region. The first of them is a component of adaptive signaling processes through stomatal closure [66]. The latter belongs to the highly conserved family of genes encoding Ca^2+^-binding protein in eukaryotes associated with abiotic/biotic stress responses in plants [67]. The two candidate genes *TraesCS2A01G534200* and *TraesCS2A01G547800* may be associated with QTL mapping of GC and ChlB content in this region. The first of them is a gene homologue of ABRE binding factor 4. One of its functions is ABA-mediated degradation of chlorophyll and senescence by activating the chlorophyll catabolism and the gene cascade of senescence [68] Another gene is a homologue of a member of the auxin-response factor gene family and is a negative regulator of fruit formation and maturation in Arabidopsis [69].

## 4. Materials and Methods

### 4.1. Genetic Material

The single chromosome substitution line S29 (YP 2A), in which chromosome 2A of the Russian cultivar S29 was substituted for the homological chromosome pair of the donor, the German cultivar YP, was developed at the Institute of Cytology and Genetics, SB RAS. The line underwent eight backcrosses. A set of 92 single chromosome recombinant double haploid (SCRDH) lines was obtained from a cross between the line S29 (YP 2A) and the recipient S29. The F_1_ plants were crossed with maize to obtain haploids [70] and, finally, double haploids. Therefore, the heterozygosity for chromosome 2A provided the genetic diversity among the SCRDH lines.

### 4.2. Growing Conditions

SCRDH S29 (YP 2A) lines were grown in three different environments. Their physiological and biochemical traits were studied under controlled conditions of climatic chamber CLF PlantMaster (CLF Plant Climatic GMBH, Wertingen, Germany), mounted in phytotron of the Siberian Institute of Plant Physiology and Biochemistry, SB RAS, Irkutsk. A soil (humus:sand:peat mixture, 1:1:1) was used as a growing substrate. The plants were grown under a 16-h photoperiod, light intensity of 350 μmol m^−2^s^−1^, day/night temperature regime of 23/16 °C, and relative humidity of 60%. Ninety-two lines and the original parental cultivars (S29 and YP) were grown in individual pots (19 cm diameter, 0.24 cm high, containing 4 kg of soil), 10 plants in each pot. The pots were kept in a climatic chamber under two irrigation regimes. Ninety-four pots were kept under normal watering and the same number of pots were kept under drought. Drought was created by watering restriction starting from the three-leaf stage until the soil water content had fallen from 60% to 30% saturation. Sowing was carried out sequentially, 25 genotypes each two weeks, so that the parameters could be measured at one stage of development. The pots were moved around the chamber every day. The experiment in climatic chamber continued until the heading of the main shoot. The yield components and developmental traits were studied in hydroponic greenhouse of the Institute of Cytology and Genetics, SB RAS, Novosibirsk. Plants were grown in bathtubs filled with keramzit (expanded clay) as a substrate. A 14-h photoperiod with day/night air temperatures of 18/20 °C before and 20/22 °C after tillering was applied. Knopp’s solution was used for plant nutrition. Five plants of each line were grown under drought and under watering during three separate greenhouse seasons: two in autumn (from September to December) and one in spring (from February to May). Drought was created by termination of irrigation at the three-leaf stage. Humidity was measured weekly using a moisture meter MG-44 (Ukraine). The dynamics of the drop in soil moisture during the greenhouse season is shown in Figure S1. It can be seen that the humidity dropped quite sharply and after 3 weeks reaching 4–5-fold differences relative to irrigation. This variant of drought can be considered as acute.

The developmental traits, stem length, peduncle length, and number of tillers were studied in the experimental field of the Institute of Cytology and Genetics SB RAS, Novosibirsk (54,845° N; 83,134° E) in 2016. The season continued from May to August; sowing date was 16 May. The Siberian climate is characterized by a lack of precipitation and moisture in the soil during the period of spring sowing and the initial development of plants. The 2016 season was characterized by a higher temperatures and uneven flow of rainwater into the soil at the first half of development of plants (Figure S1). The total volume of precipitations consisted 75% of long-term. The soils were grey forest loamy. The pre-sowing application of mineral fertilizers was carried out by calculating the active substance N60P80K60 per hectare. The lines were grown in two plots. The row length was 1 m. Twenty-five seeds were placed in each row. The distance between the rows was 20 cm. For phenotyping and statistical analysis, ten plants of each line and parental varieties were randomly selected.

### 4.3. Phenotyping

A list of studied traits in alphabetical order and their abbreviations are presented in Table A1. Their attribution to different groups is showed in Table A2.

The following physiological traits were measured using a GFS-3000 portable system for studying gas exchange and chlorophyll fluorescence (H. Walz, Effeltrich, Germany): transpiration rate (E), stomatal conductance (Gs), the rate of CO_2_ assimilation or net photosynthetic rate (A), basic chlorophyll fluorescence yield (F_0_), maximum potential quantum efficiency of PSII (Fv/Fm), effective photochemical quantum yield of PSII (Y(II)), electron transport rate (ETR), non-photochemical quenching of chlorophyll (NPQ). Water use efficiency (WUE) was calculated from the ratio A/E. Photosynthetic parameters were determined from the central part of the flag leaf on the leading tiller of eight of the ten plants per line and parental cultivars. The content of pigments in the leaves, the activity of four enzymes of the ascorbate-glutathione cycle and lipoxygenase were determined as previously described [24]. Specific activities of dehydroascorbate reductase (DHAR, EC 1.8.5.1), glutathione reductase (GR, EC 1.6.4.2), ascorbate reductase (APX, EC 1.11.1.11), superoxide dismutase (SOD, EC 1.15.1.1), catalase (CAT, EC 1.11.1.6), and lipoxygenase (LOX, EC 1.13.11.) was calculated as U/mg protein, where U is the unit of activity. The content of pigments (ChlA, ChlB, ChlA + ChlB, and Car) and the activity of enzymes were determined in three biological and three analytical replicates. One plant of each line was treated as a biological replicate. Biomass of the main shoots (SW) of 10 plants was measured.

Agronomical traits, such as days to tillering (DT), days to flowering (DF), days to wax ripening (DWR), stem length (StL), and number of productive tillers (NT) were determined under field and greenhouse conditions. Additionally, the yield components of plant were studied: spike length (SpL), number of spikelets (SpkN), main spike fertility (Fert), grain number and weight of grain at the main spike (GNmain and GW main), number and weight of grains of the secondary tillers (GNsecond and GWsecond), and total number and weight of grain from a plant (GNtotal and GWtotal), thousand grain weight (TGW). The gluten content (GC) in grains of SCRDH lines was determined for one season using hand washing (ISO 21415-1:2006) with modification for small grain samples.

The response of each trait to water-deficient conditions was assessed in the form of a tolerance index (IT, %), given by the expression Td/Tc × 100, where Td was the mean performance of the stressed plants of a given line and Tc was that of the well-watered plants. In total, 38 traits were studied, and 38 tolerance indexes were calculated.

### 4.4. Genotyping

SNP genotype profiling was done by TraitGenetics GmbH (Gatersleben, Germany; http://www.traitgenetics.com accessed on 10 August 2019). Genomic DNA was isolated from 5–7-day old seedlings. For genotyping of SCRDH lines with the Illumina Infinium 15k wheat array consisting of SNP markers mapped to the wheat genome was applied [37]. Analysis of chromosome 2A was carried out using 590 SNP markers. One hundred forty-six markers were found to be polymorphic and were used for QTL and association mapping.

### 4.5. Statistical Processing

For each line, the mean values, standard deviations, limits of values, and the ratio of the maximum value to the minimum value were calculated. Student’s t-test was used to determine significant differences between the parents of the lines. Spearman’s non-parametric correlation coefficients were calculated to reveal inter-trait correlations. Two-way ANOVA was used for analysis of the genotypic input in variability among SCRDH lines. Broad-sense heritability (H^2^) values were obtained from variance components [71]. The averaged values of traits were used for principal component analysis (PCA) and cluster analysis within PAST statistical package [72].

### 4.6. Linkage Map Construction and Association Mapping

The genetic linkage group of chromosome 2A was constructed as described in Peleg et al. [73] using the software MultiPoint (http://multiqtl.com accessed on 19 May 2021). A visualization of the map was performed by MapChart 2.2 [74]. For QTL mapping, the single-trait analysis with a 99.9% significance level within the MultiQTL program v. 2.5 was used. To determine threshold for LOD value, test with 1000 permutations was carried out.

The population structure among SCRDH lines (Q-matrix) was evaluated using the Bayesian algorithm implemented in the STRUCTURE software v. 2.3.4 [75]. The Q-matrix was calculated based on the genotyping results for 146 SNP markers. The admixture model was used to estimate the number of suspected subclusters. The number of runs was five with a burn-in length of 10,000 and Markov chain iterations of 50,000. The most likely number of clusters was calculated based on DeltaK (ΔK) statistics [76]. Marker-trait associations (MTAs) were determined based on the general linear model (GLM) using the TASSEL v. 5.2.24 software [77]. SNP markers with MAF (minor allele frequency) less than 5% were not included in the analysis. After filtering, the number of markers used for mapping was 128. A false discovery rate (FDR) of *p* < 0.01 was used to detect MTAs.

### 4.7. Bioinformatic Analysis

Here, we used the wheat assembly iwgsc_refseqv1.0 (http://wheat-urgi.versailles.inra.fr accessed on 15 January 2020). The coordinates of SNP markers on chromosome 2A were searched using BLASTn program from BLAST + package [78]. According to the gene-finding format (GFF) annotation, all sequences of the genes between the markers were extracted. Further, the functional annotation of gene homology was carried out. To do this, the synteny were identified between the wheat genes found on the target regions of chromosome 2A and genes of *Arabidopsis thaliana* using BLASTp algorithm. The extracted genes were analyzed using the associated gene ontology (GO) terms. The GO terms associated with genes homologous to wheat genes were extracted from the TAIR database (https://www.arabidopsis.org/ accessed on 15 January 2020). For each candidate gene, the domain composition was determined. The domains were described using the Hmmsearch program from the HMMER v.3 package (http://hmmer.org/ accessed on 1 March 2020) [79]. The described hidden Markov chains (Hidden Markov Model) were taken from the PFAM database (http://pfam.xfam.org/ accessed on 1 March 2020) with a threshold of *e*-value = 1 × 10^−4^.

## 5. Conclusions

In our work, the single chromosome recombinant double haploid (SCRDH) lines of bread wheat were used for the first time for mapping QTLs associated with drought tolerance. Fifty-three out of 58 QTLs associated with physiological and agronomic traits under contrasting conditions of water supply were localized in four regions of chromosome 2A, from 2.1 to 6.5 cM in length. In these regions, 39 candidate genes were identified, 18 of them were transcription factors. The functions of most of the candidate genes were associated with the hormonal signaling by means of ABA, ethylene, auxin, jasmonic, salicylic, and gibberellic acids. These phytohormones are considered to be the most important regulators of growth and development, involved in drought signaling and drought tolerance. Region 3 on the long arm of chromosome 2A, 4 cM in size, made a decisive contribution to the traits’ variation under drought. It can probably be defined as a ‘hotspot’ region for wheat drought tolerance, since it was distinguished by a high density of candidate genes participating in gene transcription, processes of photosynthesis, development and hormonal regulation of drought response. The variation in SNPs associated with agronomical and physiological traits revealed among the SCRDH S29 (YP 2A) lines is expected to provide useful information for drought related marker-assisted breeding.

## Figures and Tables

**Table 1:** Principal component analysis under two water regimes.

Principal Components	Normal Watering		Drought		Tolerance Indexes	
	Contribution to the Total Variation, %	Input of Certain Trait into Principal Component	Contribution to the Total Variation, %	Input of Certain Trait into Principal Component	Contribution to the Total Variation, %	Input of Certain Trait into Principal Component
PC1	42.8	G_S_ (−0.93) DT (0.19) LOX (0.21) WUE (0.11)	37.6	LOX (−0.93) DT (0.22) TGW (0.19) SpkN (0.12) G_S_ (−0.12)	39.6	DF (0.29), SpkN (0.28), SL (0.28), DWR (0.27), E (−0.25), G_S_ (−0.25), A (−0.25), StL (0.23), PL (0.23), GNmain (0.23), Fert (0.21), DT (0.19) SOD (0.18)
PC2	16.9	LOX (0.67) ETR (0.57) F_0_ (−0.40) G_S_ (0.19) GNmain (0.18)	27.0	DT (0.75) G_S_ (−0.59) WUE (0.13) LOX (0.17)	10.9	ChlA (0.40), ChlB (0.40), Car (0.40), ChlA+B (0.35), ETR (0.31), Fv/Fm (0.17), F_0_ (−16), WUE (0.16), E (0.15), G_S_ (0.14)
PC3	15.0	DT (0.63) GC (0.23) GNsecond (−0.38) GNtotal (−0.34) GNmain (−0.20) SpkN (0.13) DF (0.11) SOD (0.15) F_0_ (0.16) ETR (−0.12) G_S_ (0.18)	15.8	G_S_ (0.45) DT (0.23) TGW (0.59) GNsecond (−0.37) GNtotal (−0.31) LOX (0.28) GNmain (−0.19) SpkN (0.13) SOD (0.10)	7.7	ETR (0.46), Y(II) (0.45), Fv/Fm (0.40), Car (−0.26), ChB (0.24), ChlA+B (0.24), ChlA (0.22), F_0_ (−17), NT (0.19), NPQ (0.15)

Agronomical traits: DF: days to flowering; DT: days to tillering; DWR: days to wax ripening; StL: stem length (cm); PL: peduncle length (cm); NT: number of tillers; Fert: fertility; GNmain: grain number in the main spike; GNsecond: grain number in the secondary spikes; GWmain: grain weight in the main spike (g); GWsecond: grain weight in the secondary spikes (g); GNtotal: total grain number from the plant (g); GWtotal: total grain weight from the plant (g); SL: spike length (cm); SpkN: spikelets number in the main spike; TGW: 1000-grain weight of the main spike (g); GC: wet gluten content in grain (%).

**Table 2:** Comparative phenotypic parameters of S29 (YP 2A) SCRDH lines in individual clusters (in % to the general mean).

Growing Conditions	Clusters			
	1	2	3	4
Normal watering	High gas exchange: E (164), G_S_ (170), A (139), WUE (77)	Low gas exchange: E (40), G_S_ (37), A (44) Chlorophyll fluorescence: F_0_ (133), Y(II) (73), ETR (73) Yield components: GNsecond (86), GWsecond (85), GNtotal (86) LOX activity (66)	Chlorophyll content: ChlA (112), ChlB (115), ChlA+B (114) Shoot weight (82) NPQ (113) LOX activity (200)	Low gas exchange: E (74), G_S_ (72), A (83)
Drought	High gas exchange: E (134), G_S_ (135), A (135), WUE (69) Yield components: GNsecond (85), GNtotal (84), TGW (116) LOX activity (72)	Low gas exchange: E (74), G_S_ (72) LOX activity (61)	Low gas exchange: E (74), G_S_ (75), WUE (123) LOX activity (146)	Yield components: GNsecond (115), GNtotal (112) LOX activity (112)

**Table 3:** The marker-trait associations (MTAs) detected on chromosome 2A in the set of RCSDH lines for physiological, biochemical, and agronomical traits under contrasting water regimes.

Trait	Env	QTL (PVE%)	Position (cM)	Linked Marker	*p*-Value	Add
Physiological and biochemical traits						
ETR	1N	Qetr.icg-2AL (9)	78.2	BS00041816_51, Ra_c34214_1320	0.0027	−6.7
Y (II)	1N	Qyld-ph.icg-2AL (9)	78.2	BS00041816_51, Ra_c34214_1320	0.0036	0.10
ChlA	1N	QchlA.icg-2AS (10)	0.00	BobWhite_c13373_250	0.0023	0.24
ChlB	1D	QChlB.icg-2A (7)	122.5	RAC875_c27530_860	0.0090	0.13
ChlA+B	1N	QChlA_B.icg-2AS (9)	0.00	BobWhite_c13373_250	0.0031	0.32
Car	1D	QCar.icg-2A (7)	76.0	Excalibur_c47535_389, Excalibur_c28017_641	0.0089	0.12
ChlA+B/Car	1D	QChlA_B/Car 2AS (8)	66.2	IAAV2654	0.0077	−1.4
CAT	1N	Qcat.icg-2A (6)	37.5	Tdurum_contig42423_2448	0.0083	0.035
GR	1N	Qgr.icg-2A (8)	135.7	RFL_Contig5277_888	0.0066	0.08
LOX	1N	Qlox.icg-2AL.1 (8)	120.3	BS00065245_51	0.0028	−5.5
	1D	Qlox.icg-2AS.2 (4)	49.6	RAC875_rep_c78744_228, Excalibur_c92241_336	0.0069	5.1
	1D	Qlox.icg-2A.3 (4)	73.8	BobWhite_c1049_338	0.0069	−8.4
DHAR	1D	Qdhar.icg-2AL (8)	77.1	Ku_c5710_312	0.0078	0.30
SOD	1D	Qsod.icg-2AS.1 (10)	51.7	wsnp_Ex_rep_c103167_88182254, BS00076693_51	0.0012	6.9
	1D	Qsod.icg-2A.2 (8)	76.0	wsnp_CAP8_c2677_1394934	0.0063	−10.0
SW	1N	Qsw.icg-2AL (8)	77.1	Ra_c42714_1137, Ku_c5710_312	0.0026	−0.68
Agronomical traits						
DT	3N	Qdtill.icg-2A.1 (8)	76.0	Excalibur_c47535_389, Excalibur_c28017_641, wsnp_CAP8_c2677_1394934	0.0024	−1.4
	3N	Qdtill.icg-2AL.2 (7)	115.06	BS00063368_51	0.0048	−1.5
DF	3N	Qflw.icg-2AL.1 (14)	77.1	Ku_c5710_312	0.0020	−1.0
	2D	Qflw.icg-2A.1 (14)	77.1	Ra_c42714_1137	0.0002	6.4
	5	Qflw.icg-2AL.2 (12)	123.6	wsnp_Ex_c3695_6740339	0.0006	0.52
DWR	2D	Qtill.icg-2A (11)	77.1	Ra_c42714_1137	0.0008	10.2
StL	3N	Qstl.icg-2AL.1 (8)	88.1	GENE-1381_132	0.0080	9.4
	4N	Qstl.icg-2AL.2 (10)	77.1	Ku_c5710_312	0.0087	3.3
	4D	Qstl.icg-2A.3 (8)	49.6	RAC875_rep_c78744_228	0.0084	5.1
	2D	Qstl.icg-2AL.4 (8)	78.2	BS00041816_51, Ra_c34214_1320	0.0046	10.5
	2D	Qstl.icg-2A.2 (7)	77.1	Ra_c42714_1137	0.0078	10.1
	5	Qstl.icg-2AL.4 (15)	78.2	Ra_c34214_1320, BS00041816_51	0.0002	2.2
SpL	3N	Qsl.icg-2AS.1 (9)	45.2	Tdurum_contig42153_5854, RAC875_rep_c111906_144	0.0028	−0.76
	4N	Qsl.icg-2AS.2 (8)	0.00	BobWhite_c13373_250	0.0041	−3.8
GNmain	3N	Qgrms.icg-2SA (8)	45.2	Tdurum_contig42153_5854, RAC875_rep_c111906_144	0.0039	−0.4
PL	4N	Qped.icg-2A (10)	73.8	BobWhite_c1049_338	0.0019	3.5
	5	Qped.icg-2A (8)	73.8	BS00078116_51, BobWhite_c1049_338	0.0069	−1.6
TGW	3N	Qtgw-2AS.1 (11)	68.4	wsnp_Ex_c15822_24204224, BS00083146_51	0.0008	8.6
	3N	Qtgw-2A.2 (7)	77.1	Ra_c42714_1137	0.0086	−5.4
	4N	Qtgw-2A.3 (6)	73.8	BobWhite_c1049_338	0.0080	0.78
	3D	Qtgw.icg-2A.2 (11)	77.1	Ra_c42714_1137	0.0015	−0.45
	4D	Qtgw.icg-2AL.2 (10)	77.1	Ku_c5710_312	0.0053	−3.0
SpkN	3D	Qspln.icg-2A (7)	77.1	Ra_c42714_1137	0.0064	−1.7
GWmain	3D	Qgwms.icg-2A (11)	76.0	Excalibur_c47535_389, Excalibur_c28017_641	0.0013	−0.29
GNsecond	4D	Qgnsec.icg-2AS (7)	66.2	IAAV2654, Kukri_c1728_1876	0.0087	−0.68
GNtotal	3D	Qtotgn.icg-2A (11)	77.1	Ra_c42714_1137	0.0015	−0.47
GC	3N	Qglucont.icg-2A.1 (8)	120.3	BS00065245_51	0.0045	1.7
	3D	Qglucont.icg-2A.2 (8)	67.2	RAC875_c17787_274	0.0045	1.7
Tolerance indices						
IT-A	1	QIT-Aphot.icg-2AL (13)	771	Ku_c5710_312	0.0002	171
IT-E	1	QIT-Ephot.icg-2AL (10)	77.1	Ra_c42714_1137	0.0019	221
IT-Gs	1	QIT-Gsphot.icg-2AL (10)	77.1	Ra_c42714_1137	0.0020	223
IT-ETR	1	QIT-etr.icg-2AL (10)	78.2	Ra_c34214_1320, BS00041816_51	0.0017	30
IT-Y(II)	1	QIT-yld-ph.icg-2AL (10)	78.2	Ra_c34214_1320, BS00041816_51	0.0018	30
IT-DT	3	QIT-dtill.icg-2AL (6)	123.6	wsnp_Ex_c3695_6740339	0.0082	1.8
IT-StL	3	QIT-stl.icg-2A (10)	111.9	TA004785-1734, Excalibur_c18514_238	0.0021	11
IT-SpL	4	QIT-sl.icg-2AS (6)	0.00	BobWhite_c13373_250	0.0045	−4.1
IT-SpkN	3	QIT-spln.icg-2AS (11)	48.5	CAP11_c2293_200	0.0011	6.7
IT-GWmain	3	QIT-wms.icg-2AL (10)	77.1	Ku_c5710_312	0.0096	−20
IT-TGW	3	QIT-tgw.icg-2AL (11)	77.1	Ku_c5710_312	0.0074	8.3
IT-GNsecond	3	QIT-gnsec.icg-2A (9)	77.1	Ra_c42714_1137	0.0040	24
IT-GWsecond	3	QIT-gwst.icg-2AL (11)	120.3	BS00065245_51, Tdurum_contig53038_714	0.0011	9.2
IT-GNtotal	3	QIT-totgn.icg-2A (9)	122.5	RAC875_c27530_860	0.0045	12

Env: environment; 1: climatic chamber; 2–4: greenhouse seasons; 5: field; N: normal watering; D: drought; PVE: explained phenotypic variance; QTL: position in cM from the top of the linkage group. The loci with a significance *p*-value < 0.01 and < 0.001 are presented. Additive effects are presented in absolute units of the traits. Abbreviations as in Table 1.

**Table 4:** Distribution of genes in the regions of chromosome 2A containing QTL clusters.

Chromosome Region (cM)			Gene Number			
N	Position	Length	In the Region	per cM	Candidate	Including Candidate TF
1	45.2–51.7	6.5	132	20	4	1
2	66.2–68.3	2.1	189	90	9	7
3	73.8–78.1	4.3	523	122	17	5
4	117.6–123.0	5.4	250	46	9	4
	Total gene number		1094	-	39	18

## Data Availability

The study did not report any data.

## Supplementary Materials

The following are available online at https://www.mdpi.com/article/10.3390/plants10051023/s1, Figure S1: PCA biplot for phenotypic variability of physiological, biochemical and agronomic traits among 92 SCRDH lines S29 (YP 2A) under normal watering (A) and drought (B), Figure S2: Clustering of SCRDH lines based on phenotypic variability according to 38 parameters under normal watering and drought, Figure S3: The genetic structure of 92 SCRDH lines S29 (YP 2A) based on 146 SNPs. Each SCRDH line is represented by a single vertical line that is partitioned into Q colored segments (*Q* = 5) in the *x*-axis (Qmatrix). The *y*-axis illustrated the *Q*-value, Figure S4: A. Dynamics of humidity level of substrate (%) in bathtubs with normal irrigation (grey) and under drought condition (black); termination of irrigation at the three-leaf stage is indicated by arrow. B, C. Ten-day dynamics of temperature and precipitation in the field, May–August 2016, Novosibirsk, Table S1: Broad-sense heritability and average meanings of phenotypic traits in the S29 (YP 2A) SCRDH lines and their parents under two water regimes, Table S2: Tolerance indices of the C29 (YP 2A) SCRDH lines and their parents. Table S3: Trait correlations under normal watering and drought, Table S4: Candidate genes and their annotations.

## Appendix A

**Table A1:** List of abbreviations.

A	photosynthetic rate
APX	ascorbate peroxidase
Car	carotenoids
CAT	catalase
ChlA, B	chlorophyll A, B
DF	days to flowering
DHAR	dehydroascorbate reductase
DT	days to tillering
DWR	days to wax ripening
E	transpiration rate
ETR	maximum electron transport rate
Fert	spike fertility as spikelet number/spike length
Fv/Fm	maximum quantum yield of PSII photochemistry
F_0_	basic chlorophyll fluorescence yield
GC	wet gluten content in grain
GNmain	grain number in the main spike
Gnsecond	grain number in the secondary spikes
GNtotal	total grain number from the plant
GR	glutathione reductase
Gs	stomatal conductance
Gwmain	grain weight in the main spike
Gwsecond	grain weight in the secondary spikes
GWtotal	total grain weight from the plant
LOX	lipoxygenase
NPQ	non-photochemical quenching
NT	number of tillers
PL	peduncle length
SL	spike length
SOD	superoxide dismutase
SpkN	spikelets number in the main spike
StL	stem length
SW	fresh weight of the main shoot
TGW	1000-grain weight as grain weight/grain number
WUE	water use efficiency as net photosynthesis/transpiration
Y(II)	effective photochemical quantum yield of photosystem II

**Table A2:** Distribution of traits by physiological, biochemical and agronomical groups.

Physiological and Biochemical Traits				Agronomic Traits	
Photosynthesis	Chlorophyll Fluorescence	Leaf Pigments	Enzymes Activity	Developmental	Yield Components
A, E, Gs, WUE, SW	F0, Fv/Fm, Y(II), ETR, NPQ	ChlA, ChlB, Car, ChlA+B, ChlA+B/Car	APX, DHAR, SOD, LOX, GR, CAT	DT, DF, DWR	StL, NT, SL, PL, Fert, GNmain, GWmain, GNsecond, GWsecond, GNtotal, GWtotal, TGW, GC
