# Supplementary material for: Regions of Chromosome 2A of Bread Wheat (Triticum aestivum L.) Associated with Variation in Physiological and Agronomical Traits under Contrasting Water Regimes
Source: Plants (Basel). 2021 May 20;10(5):1023. doi: 10.3390/plants10051023 (PMC8161357; doi:10.3390/plants10051023)
Supplement: Supplementary file 1 [file plants-10-01023-s001.zip › plants-1210017-Supplementary/Supplementary/Figure S1.pdf]

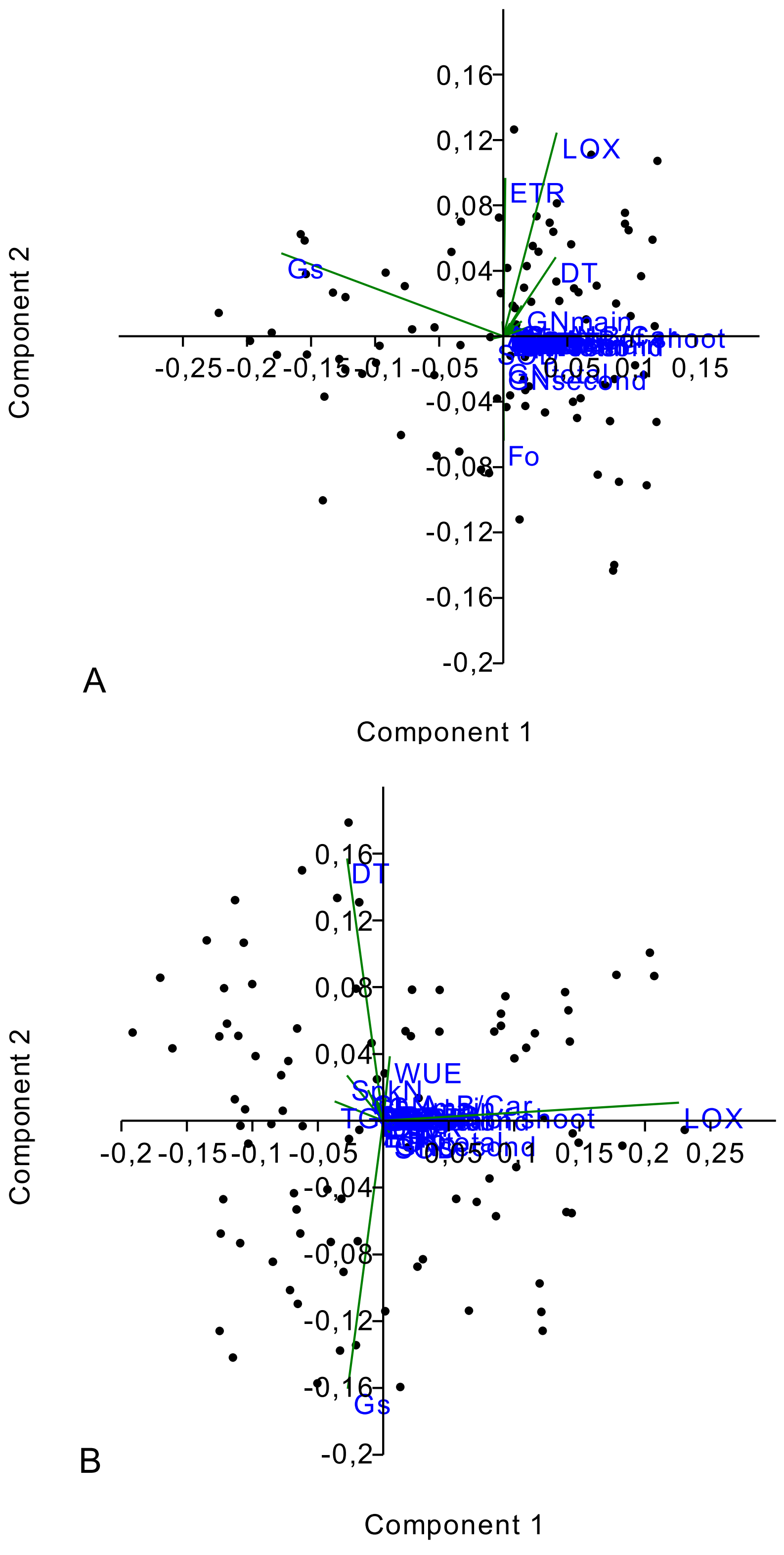

Figure S1. PCA biplot for phenotypic variability of physiological, biochemical and agronomic traits among 92 SCRDH lines S29 (YP 2A) under normal watering (A) and drought (B).
