# Supplementary material for: Regions of Chromosome 2A of Bread Wheat (Triticum aestivum L.) Associated with Variation in Physiological and Agronomical Traits under Contrasting Water Regimes
Source: Plants (Basel). 2021 May 20;10(5):1023. doi: 10.3390/plants10051023 (PMC8161357; doi:10.3390/plants10051023)
Supplement: Supplementary file 1 [file plants-10-01023-s001.zip › plants-1210017-Supplementary/Supplementary/Figure S4.pdf]

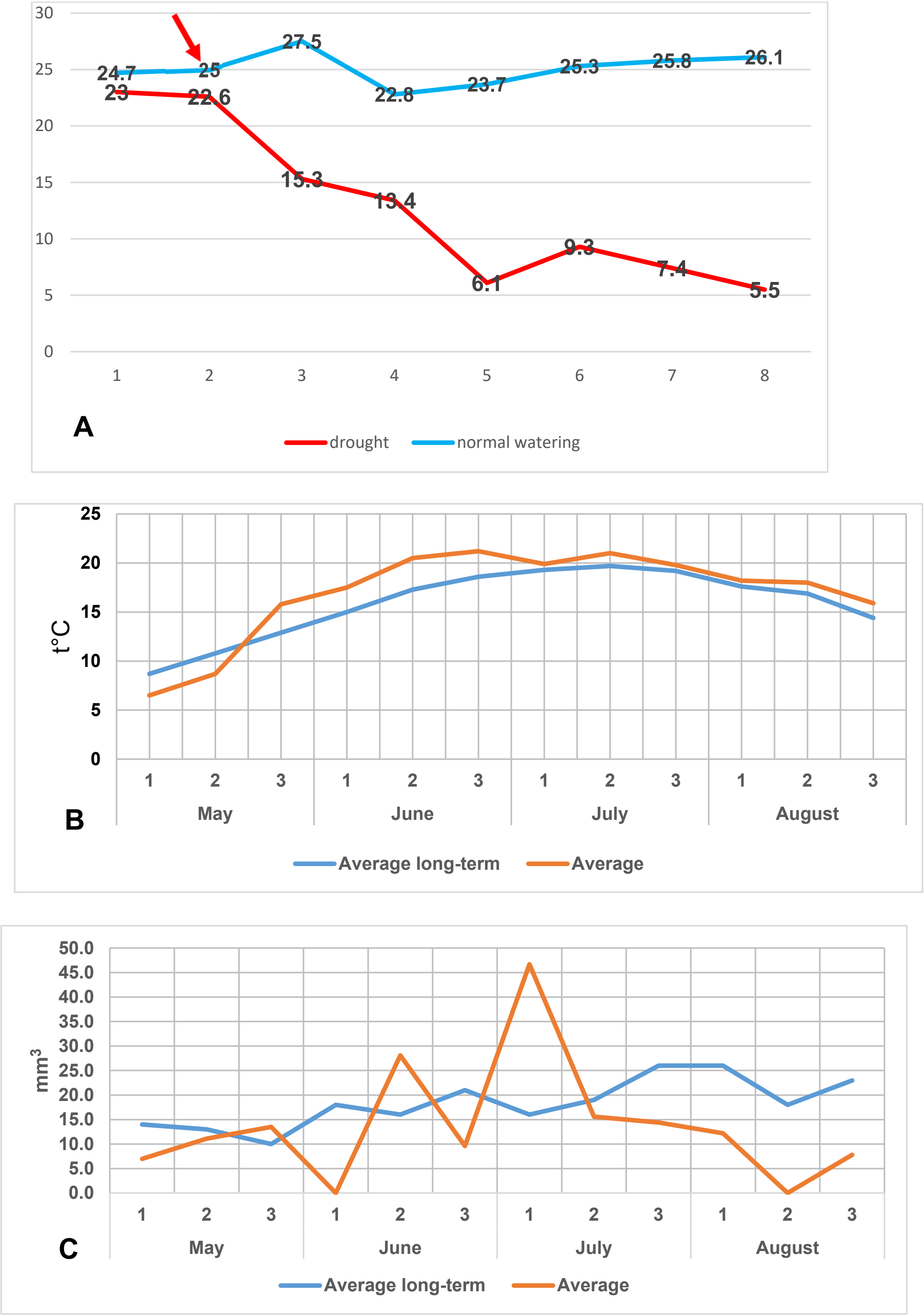

Figure S4. A. Dynamics of humidity level of substrate (%) in bathtubs with normal irrigation (grey) and under drought condition (black); termination of irrigation at the three-leaf stage is indicated by arrow. B, C. Ten-day dynamics of temperature and precipitation in the field, May-August, 2016, Novosibirsk.
