# Supplementary material for: Regions of Chromosome 2A of Bread Wheat (Triticum aestivum L.) Associated with Variation in Physiological and Agronomical Traits under Contrasting Water Regimes
Source: Plants (Basel). 2021 May 20;10(5):1023. doi: 10.3390/plants10051023 (PMC8161357; doi:10.3390/plants10051023)
Supplement: Supplementary file 1 [file plants-10-01023-s001.zip › plants-1210017-Supplementary/Supplementary/Table S1.pdf]

Table S1. Broad-sense heritability and average meanings of phenotypic traits in the S29 (YP 2A) SCRDH lines and their parents under two water regimes.

Traits	Parents		Recombinant lines			
	S29	YP	Average±SD	Limits	max/min	H^2
Normal watering						
DT	23.9	21.3*	19.0±0.9	17.5-23.2	1.3	0.25
DF	43.5	46.5**	41.2±1.1	38.9-44.7	1.2	0.56
DWR	68.5	73.0*	66.2±2.1	57.9-71.6	1.2	0.48
StL	86.2	72.8*	87.8±3.6	76.5-96.3	1.3	0.23
PL	37.7	32.3**	37.4±1.9	31.1-41.2	1.3	0.44
NT	3.4	3.0	4.4±0.5	3.2-5.6	1.8	0.05
SL	7.1	9.0***	7.5±0.4	6.4-8.3	1.3	-
SpkN	13.0	17.3***	13.8±0.6	12.4-15.1	1.2	0.15
GNmain	25.3	29.3	28.8±2.3	22.3-33.6	1.5	0.15
GWmain	1.0	1.1	1.1±0.1	0.9-1.5	1.7	0.17
Fert	1.9	1.7	1.5±0.1	0.87-1.73	2.0	0.10
GNsecond	57.8	65.3	97.9±16.7	62.2-139.1	2.2	0.13
GWsecond	1.8	2.1	3.4±0.7	2.0-5.0	2.5	0.16
GNtotal	83.0	94.6	113.5±17.7	73.3-154.6	2.1	0.13
GWtotal	2.8	3.1	4.6±0.8	2.9-6.4	2.2	0.16
TGW	39.5	36.0	39.4±2.4	32.8-44.5	1.4	0.20
SW	5.0	6.2***	4.4±0.5	3.5-5.8	1.7	0.31
E	0.39	0.38	0.5±0.3	0.1-1.2	12.1	0.59
Gs	27.3	27.1	38.6±20.4	6.4-94.0	14.6	0.59
A	2.0	2.0	2.3±0.8	0.6-4.2	6.7	0.68
WUE	5.7	6.7	5.2±1.3	2.7-8.5	3.2	0.34
F_0	93.0	87.0	95.3±20.5	63.3-184.3	2.9	0.61
Fv/Fm	0.8	0.8	0.7±0.1	0.5-0.9	1.7	0.82
Y(II)	0.5	0.5	0.5±0.1	0.3-0.6	2.4	0.61
ETR	34.9	34.7	33.0±4.2	16.3-39.8	2.4	0.61
NPQ	0.3	0.5	0.4±0.1	0.2-0.6	3.3	0.31
ChlA	2.0	1.98	1.9±0.3	1.0-2.9	2.8	0.54
ChlB	1.0	1.0	0.9±0.2	0.59-1.27	2.2	0.46
ChlA+B	3.1	2.8	2.8±0.1	1.7-4.1	2.4	0.54
Car	0.43	0.39*	0.4±0.1	0.3-0.6	2.2	0.50
ChlA+B/Car	7.6	7.3	7.1±0.8	5.7-9.8	1.7	0.38
SOD	49.8	45.5	49.4±6.7	29.5-98.6	3.3	0.18
APX	1.64	1.94**	1.7±0.4	0.8-2.8	3.3	0.47
DHAR	1.41	1.33	1.1±0.4	0.4-2.2	5.6	0.44
GR	0.21	0.17	0.2±0.1	0.1-0.8	9.5	0.35
CAT	0.16	0.13	0.1±0.0	0.1-0.3	6.0	0.43
LOX	16.4	11.0**	12.5±5.6	4.0-30.3	7.5	0.66

GC	38.5	34.5	35.3±2.1	31.0-39.8	1.28	-
			Drought			
DT	19.8	18.5	21.6±1.4	18.1-25.3	1.4	0.40
DF	43.8	47.8**	41.2±1.3	38.5-44.0	1.1	0.55
DWR	70.8	73.2*	65.9±3.2	58.7-72.4	1.2	0.38
StL	85.8	76.4*	83.2±3.5	75.4-91.8	1.2	0.15
PL	36.6	32.3*	35.7±2.2	29.3-47.1	1.6	0.20
NT	2.8	2.6	4.0±0.4	3.1-5.4	1.7	0.19
SL	7.4	9.2***	7.4±0.3	6.6-8.3	1.3	0.16
SpkN	14.1	18.6***	13.9±0.7	12.5-15.5	1.2	0.22
GNmain	25.3	33.3*	28.1±2.2	23.0-33.0	1.4	0.13
GWmain	0.82	0.87	0.8±0.1	0.6-1.1	1.9	0.23
Fert	1.8	1.9	2.0±0.2	1.6-2.4	1.5	0.09
GNsecond	39.5	49.0	52.1±10.6	22.9-75.3	3.3	0.23
GWsecond	1.0	0.9	1.2±0.3	0.6-2.7	4.2	0.23
GNtotal	64.8	76.9	79.7±12.9	32.5-108.3	3.3	0.20
GWtotal	1.8	1.8	2.0±0.3	1.5-3.3	2.2	0.29
TGW	32.8	26.3**	28.1±4.2	21.3-38.7	1.8	0.57
SW	2.7	3.2***	2.7±0.3	2.2-3.6	1.6	0.50
E	0.6	0.55	0.6±0.3	0.1-1.4	9.9	0.62
Gs	39.6	38.9	45.4±20.1	10.1-108.0	10.7	0.61
A	2.6	2.28	2.8±0.7	1.1-4.6	4.2	0.67
WUE	5.6	4.6	5.5±2.0	2.3-11.9	5.2	0.62
F_0	78.5	84.8	75.6±9.2	57.3-105.2	1.8	0.51
Fv/Fm	0.8	0.8	0.8±0.0	0.75-0.81	1.1	0.47
Y(II)	0.5	0.5	0.5±0.0	0.5-0.6	1.3	0.20
ETR	35.6	33.7	35.6±1.8	30.1-39.3	1.3	0.20
NPQ	0.4	0.4	0.4±0.1	0.2-0.7	3.5	0.57
ChlA	2.3	1.8***	2.2±0.3	1.3-3.2.6	2.5	0.42
ChlB	1.09	0.98	1.0±0.2	0.7-1.4	2.0	0.61
ChlA+B	3.4	2.8**	3.3±0.5	2.0-4.5	2.0	0.45
Car	0.46	0.37**	0.4±0.1	0.2-0.6	2.6	0.51
ChlA+B/Car	8.1	8.0	7.8±0.9	6.2-11.5	1.9	0.65
SOD	50.5	50.1	51.8±6.0	32.8-72.7	2.2	0.15
APX	1.66	1.41*	1.7±0.5	0.9-4.1	4.6	0.59
DHAR	0.71	0.61	1.2±0.4	0.5-2.3	4.6	0.39
GR	0.30	0.31	0.3±0.1	0.1-0.7	7.3	0.25
CAT	0.14	0.13	0.1±0.1	0.1-0.4	6.7	0.26
LOX	21.6	16.9**	17.1±6.5	4.7-33.3	7.1	0.63
GC	39.0	37.0	37.9±2.7	30.5-41.8	1.38	-

*, **, *** means differed significantly at P<0.05, 0.01, and 0.001, respectively.

Physiological and biochemical traits: A: photosynthetic rate (μmol m-2 s-1); Car: carotenoids; ChlA,B: chlorophyll A,B (mg/g of dry leaf weight); E: transpiration rate (mmol m-2 s-1); WUE: wa-ter use efficiency as net photosynthesis/transpiration; ETR: maximum electron transport rate (photon m–2 sec–1); F0: basic chlorophyll fluorescence yield; Fv/Fm: maximum quantum yield of PSII photochemistry; NPQ: non-photochemical quenching; Gs: stomatal conductance (mol m-2 s-1); SW: fresh weight of the main shoot (g); Y(II): effective photochemical quantum yield of photosys-tem II; CAT, DHAR, GR, SOD, LOX: activity of catalase, dehydroascorbate reductase, glutathione reductase, superoxide dismutase and lipoxygenase (U/mg protein).
Agronomical traits: DF: days to flowering; DT: days to tillering; DWR: days to wax ripening; StL: stem length (cm); PL: peduncle length (cm); NT: number of tillers; Fert: fertility; GNmain: grain number in the main spike; GNsecond: grain number in the secondary spikes; GWmain: grain weight in the main spike (g); GWsecond: grain weight in the secondary spikes (g); GNtotal: total grain number from the plant (g); GWtotal: total grain weight from the plant (g); SpL: spike length (cm); SpkN: spikelets number in the main spike; StL: stem length (cm); TGW: 1,000-grain weight of the main spike (g); GC: wet gluten content in grain (%).
