# Supplementary material for: Regions of Chromosome 2A of Bread Wheat (Triticum aestivum L.) Associated with Variation in Physiological and Agronomical Traits under Contrasting Water Regimes
Source: Plants (Basel). 2021 May 20;10(5):1023. doi: 10.3390/plants10051023 (PMC8161357; doi:10.3390/plants10051023)
Supplement: Supplementary file 1 [file Figure S2.pdf › plants-1210017-Supplementary/Supplementary/Figure S2.pdf]

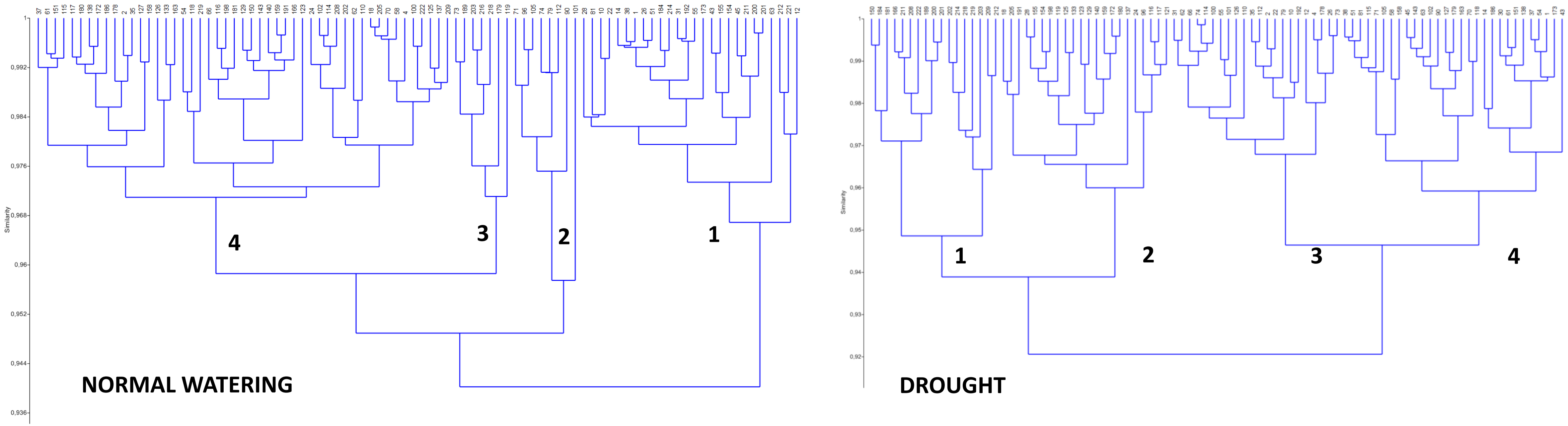

Figure S2. Clustering of SCRDH lines based on phenotypic variability according to 38 parameters under normal watering and drought.
