# Supplementary material for: Regions of Chromosome 2A of Bread Wheat (Triticum aestivum L.) Associated with Variation in Physiological and Agronomical Traits under Contrasting Water Regimes
Source: Plants (Basel). 2021 May 20;10(5):1023. doi: 10.3390/plants10051023 (PMC8161357; doi:10.3390/plants10051023)
Supplement: Supplementary file 1 [file plants-10-01023-s001.zip › plants-1210017-Supplementary/Supplementary/Table S2.pdf]

**Table S2.** Tolerance indices of the C29 (YP 2A) SCRDH lines and their parents.

| | Parents | | SCRDH lines | | |
|---|---|---|---|---|---|
| Traits | S29 | YP | Average ± SD | Limits | max/min |
| DT | 107 | 109 | 115±10 | 91-141 | 1.6 |
| DF | 91 | 101 | 103±4 | 91-112 | 1.2 |
| DWR | 114 | 107 | 101±7 | 84-117 | 1.4 |
| StL | 100 | 102 | 94±4 | 86-104 | 1.2 |
| PL | 96 | 101 | 95±6 | 86-131 | 1.5 |
| NT | 82 | 90 | 92±19 | 77-157 | 2.0 |
| SL | 104 | 103 | 100±6 | 86-115 | 1.3 |
| SpkN | 109 | 110 | 102±5 | 85-114 | 1.3 |
| GNmain | 99 | 114 | 98±11 | 72-141 | 2.0 |
| GWmain | 83 | 84 | 70±11 | 50-115 | 2.3 |
| Fert | 91 | 104 | 97±11 | 75-135 | 1.8 |
| GNsecond | 68 | 75 | 55±13 | 25-105 | 4.2 |
| GWsecond | 59 | 51 | 36±10 | 17-71 | 4.2 |
| GNtotal | 78 | 88 | 64±12 | 39-114 | 2.9 |
| GWtotal | 69 | 64 | 44±10 | 26-78 | 3.0 |
| TGW | 85 | 73 | 72±11 | 52-103 | 2.0 |
| SW | 52 | 57 | 62±8 | 46-88 | 1.9 |
| E | 68 | 144 | 157±133 | 18-710 | 39.4 |
| Gs | 66 | 143 | 164±149 | 16-795 | 49.7 |
| A | 84 | 115 | 142±90 | 44-566 | 12.9 |
| WUE | 123 | 69 | 113±51 | 40-306 | 7.7 |
| $F_0$ | 84 | 98 | 82±16 | 47-121 | 2.6 |
| Fv/Fm | 103 | 102 | 108±11 | 92-153 | 1.7 |
| Y(II) | 102 | 97 | 110±18 | 82-208 | 2.5 |
| ETR | 102 | 97 | 110±18 | 82-211 | 2.6 |
| NPQ | 113 | 84 | 108±49 | 35-244 | 7.0 |
| ChlA | 111 | 99 | 123±29 | 70-232 | 3.3 |
| ChlB | 105 | 101 | 118±25 | 73-194 | 2.7 |
| ChlA+B | 109 | 100 | 121±26 | 71-208 | 2.9 |
| Car | 107 | 95 | 110±24 | 65-184 | 2.8 |
| ChlA+B/Car | 107 | 107 | 111±15 | 84-144 | 1.7 |
| SOD | 101 | 111 | 106±16 | 73-152 | 2.1 |
| APX | 107 | 77 | 110-44 | 40-324 | 8.1 |
| DHAR | 49 | 49 | 125±58 | 37-354 | 9.6 |
| GR | 150 | 185 | 131±60 | 15-346 | 23.1 |
| CAT | 88 | 100 | 107±44 | 38-257 | 6.6 |
| LOX | 131 | 153 | 164±93 | 30-457 | 15.2 |
| GC | 99 | 93 | 108±8 | 83-131 | 1.6 |

Abbreviations as in Table S1.
